# Diversification of the P genome among *Agropyron* Gaertn. (Poaceae) species detected by FISH

**DOI:** 10.3897/CompCytogen.v11i3.13124

**Published:** 2017-08-03

**Authors:** Yan Zhao, Jihong Xie, Quanwen Dou, Junjie Wang, Zhong Zhang

**Affiliations:** 1 College of Grassland, Resource and Environmental Science, Inner Mongolia Agricultural University, Hohhot, 010011, China; 2 Key Laboratory of Grassland Resources (IMAU), Ministry of Education, Hohhot, 010011, China; 3 Key Laboratory of Forage Cultivation, Processing and High Efficient Utilization of Ministry of Agriculture, Hohhot, 010011, China; 4 Grassland Research Institute, Chinese Academy of Agricultural Sciences, Hohhot, 010010, China; 5 Key Laboratory of Crop Molecular Breeding, Qinghai Province, Northwest Institute of Plateau Biology, Chinese Academy of Sciences, Xining 810001, China

**Keywords:** *Agropyron*, FISH, P genome, rDNA, repetitive sequence

## Abstract

The genomes of five *Agropyron* Gaertner, 1770 species were characterized using all potential di- or trinucleotide simple sequence repeat (SSR) motifs and four satellite DNA repeats as fluorescence in situ hybridization (FISH) probes. The sites of 5S and 45S rDNA were relatively conserved among the diploid and tetraploid species. A number of sites for the dinucleotide SSRs AC, AG, and pSc119.2 was detected in all investigated species except *A. mongolicum* Keng, 1938. Several different trinucleotide SSRs were identified in different tetraploid species. All *Agropyron* species were suggested to include the basic P genome, although genome differentiation was still observed. The P genome of *A. mongolicum* was distinct from that of the diploid *A. cristatum* (Linnaeus, 1753) Gaertner, 1770. and other tetraploid species, with no hybridizations for AC, AG, or pSc119.2 observed. This finding supports designation of the P genomes of *A. cristatum* and *A. mongolicum* as P^c^ and P^m^, respectively. An exceptional 5S rDNA site revealed in one set of homoeologous chromosomes strongly supports the allopolyploid origin of *A. desertorum* (Fischer ex Link, 1821) Schultes, 1824. However, the diploid donors to *A. desertorum* need further investigation. Similarly, the unique FISH pattern of a pair of 5S rDNA-carrying chromosomes was indicative of a potential allopolyploid origin for *A. fragile* (Roth, 1800) Candargy, 1984. The conserved distribution of 5S and 45S rDNA suggests *A. cristatum* (4x) and *A. michnoi* Roshevitz, 1929 are closely related. Two forms of B chromosomes were identified among individuals *A. mongolicum* and *A. desertorum* plants.

## Introduction

The genus *Agropyron* Gaertner, 1770, also referred to as the crested wheatgrass complex, includes 10–15 species (Asay and Jensen 1996). The genus is distributed in Eurasia and comprises a series of diploid (2n = 14), tetraploid (2n = 28), and hexaploid (2n = 42) species (Löve 1982, 1984, Dewey 1984). The diploid species are less common and distributed from Europe to Mongolia, whereas tetraploids commonly occur in central Europe, the Middle East, and central Asia. Hexaploids are rare in Turkey and Iran (Dewey and Asay 1982). The spike morphology is important for identification for the species, and varies in a continuous fashion from broad, pubescent, pectinate spikes to narrow, linear, glabrous spikes (Dewey and Asay 1982). The species taxonomy is confounded by overlapping geographic distributions and high cross-compatibility among species. The taxonomy of *Agropyron* spp. has been characterized as “a multitude of taxonomic binomials” (Dewey and Pendese 1967). Although morphologically diverse, all crested wheat grasses are considered to share the same basic genome; this was previously termed the C genome and is currently referred to as the P genome (Dewey and Pendese 1967).

Inter-specific differentiation in *Agropyron* has been extensively studied using morphology, cytology and molecular markers. *Agropyron mongolicum* Keng, 1938 is a diploid species indigenous to northern China. It is distinguished from the diploid species *A. cristatum* (Linnaeus, 1753) Gaertner, 1770 based on its narrow, linear spikes (Dewey 1981). Hsiao et al. (1986, 1989) revealed *A. cristatum* and *A. mongolicum* share the same basic P genome but differ as a result of structural rearrangement of some chromosomes. These findings were based on a karyotype analysis and meiotic analysis of interspecific hybrids. Data from amplified fragment length polymorphism (AFLP) markers showed that *A. mongolicum* clusters separately from other *Agropyron* species, including *A. cristatum* and *A. fragile* (Roth, 1800) Candargy, 1984 (Mellish et al. 2002). These findings suggest that the P genomes of *A. cristatum* and *A. mongolicum* should be designated “P^c^” and “P^m^”, respectively (Mellish et al. 2002). The origin of *A. desertorum* (Fischer ex Link, 1821) Schultes, 1824 has been extensively discussed. Taylor and McCoy (1973) concluded that *A. desertorum* originated from amphidiploids between *A. imbricatum* Roemer & Schultes, 1817 and *A. pectiniforme* Roemer & Schultes, 1817 on the basis of chromatographic and karyotypic analysis. Both *A. imbricatum* and *A. pectiniforme* have been reclassified subsequently as subspecies of *A. cristatum* (Tzvelev 1976). On the basis of morphology and meiotic relationship in hybrids between *A. mogolicum* × *A. cristatum* amphiploid and *A. desertorum*, Asay et al. (1992) suggested that the ancestral germplasm of *A. desertorum* was derived from hybridization between *A. mongolicum* and *A. cristatum*, followed by chromosome doubling. In the same study, the authors further proposed that *A. fragile* is an autotetraploid derivative of *A. mongolicum*, and that variants of *A. desertorum* could be generated from hybrids between *A. fragile* and tetraploid *A. cristatum* (Asay et al. 1992). Molecular data from AFLP markers suggest that *A. desertorum* is an alloploid incorporating the P genomes of *A. mongolicum* and *A. cristatum*, and thus the genome of *A. desertorum* could be designated “P^c^P^c^P^m^P^m^” (Mellish et al. 2002). However, Vogel et al. (1999) concluded that the genome length of *A. desertorum* was more consistent with that of an autoploid of *A. cristatum* than that of an allotetraploid between *A. cristatum* and *A. mongolicum*. Moreover, Che et al. (2015) distinguished populations of *A. desertorum*, *A. mongolicum* and *A. michnoi* Roshev. from populations of *A. cristatum* by means of principal coordinate analysis. The results suggest that *A. desertorum*, *A. mongolicum* and *A. michnoi* Roshevitz, 1929 are derivative species of *A. cristatum* that share the same basic genome as the ancestral species.

Fluorescence *in situ* hybridization (FISH) is a powerful tool for characterization of genome composition based on a few repetitive sequences. Species-level phylogenies across species can be derived from repeat-based comparative FISH karyotyping (Jiang and Gill 2006, Heslop-Harrison and Schwarzacher 2011). Comparative FISH may allow to detect genome differentiation between closely related species (Carmona et al. 2013, Dou et al. 2016).

The genomes of Triticeae species are large, of which 75% of the genome comprises repetitive sequences (Flavell et al. 1974, 1977). The genome composition in Triticeae species can be characterized using different microsatellite or satellite repetitive sequences via FISH (Cuadrado et al. 2013; Dou et al. 2016). In the present study, comparative FISH was conducted using all potential dinucleotide and trinucleotide simple sequence repeats (SSRs), 5S and 45S ribosomal DNA (rDNA), pSc119.2, and pAs1 repeats on mitotic chromosomes of five *Agropyron* species. The objective was to examine the genome differentiation among species within the genus *Agropyron* and infer genetic relationships among the investigated *Agropyron* species.

## Material and methods

### Plant material

Six accessions belonging to *A. mongolicum*, *A. cristatum*, *A. desertorum*, *A. fragile*, and *A. michnoi* were used in this study (Table 1). Seeds of each accession were randomly selected for cytogenetic investigation.

**Table 1.** Plant materials used in the study.

No.	Species	Provenance
1	*Agropyron mongolicum* Keng, 1938	Inner Mongolia, China
2	*Agropyron cristatum* (2x) (Linnaeus, 1753) Gaertner, 1770 ‘Fairway’	USA
3	*Agropyron cristatum* (4x) (Linnaeus, 1753) Gaertner, 1770	Qinghai, China
4	*Agropyron desertorum* (Fischer ex Link, 1821) Schultes, 1824 ‘Nordan’	USA
5	*Agropyron fragile* (Roth, 1800) Candargy, 1984	Inner Mongolia, China
6	*Agropyron michnoi* Roshevitz, 1929	Inner Mongolia, China

### Slide preparation

The seeds were germinated on moist filter paper in Petri dishes at room temperature. Root tips of of 1–2 cm length were excised and pretreated with nitrous oxide at a pressure of 8 atm for 2 h at room temperature following the method of Kato (1999). Subsequently, the root tips were fixed in ethanol–glacial acetic acid (3:1, v/v) for at least 30 min at room temperature. Each root tip was squashed in a drop of 45% acetic acid.

### DNA probes and labelling

All potential dinucleotide and trinucleotide SSRs and four satellite DNA sequences, namely pAs1 (Rayburn and Gill 1986), pSc119.2 (Bedbrook et al. 1980), 5S and 45S rDNA, were used to generate DNA probes. The procedure for labeling the above sequences was performed in accordance with the method of Dou et al. (2016).

### FISH and microphotometry

The FISH experiments were conducted following the method of Dou et al. (2009; 2016). The images were captured with a Photometrics CoolSNAP CCD camera (Roper Scientific, Trenton, NJ, USA) using a fluorescence microscope (model DM R HC, Leica Microsystems, Wetzlar, Germany) and processed with the Meta imaging system (Universal Imaging Corporation, West Chester, PA, USA). Finally, the images were adjusted with Adobe Photoshop 6.0 for contrast and background optimization.

## Results

### Karyotype features of the P genome among species

**Figure 1.**
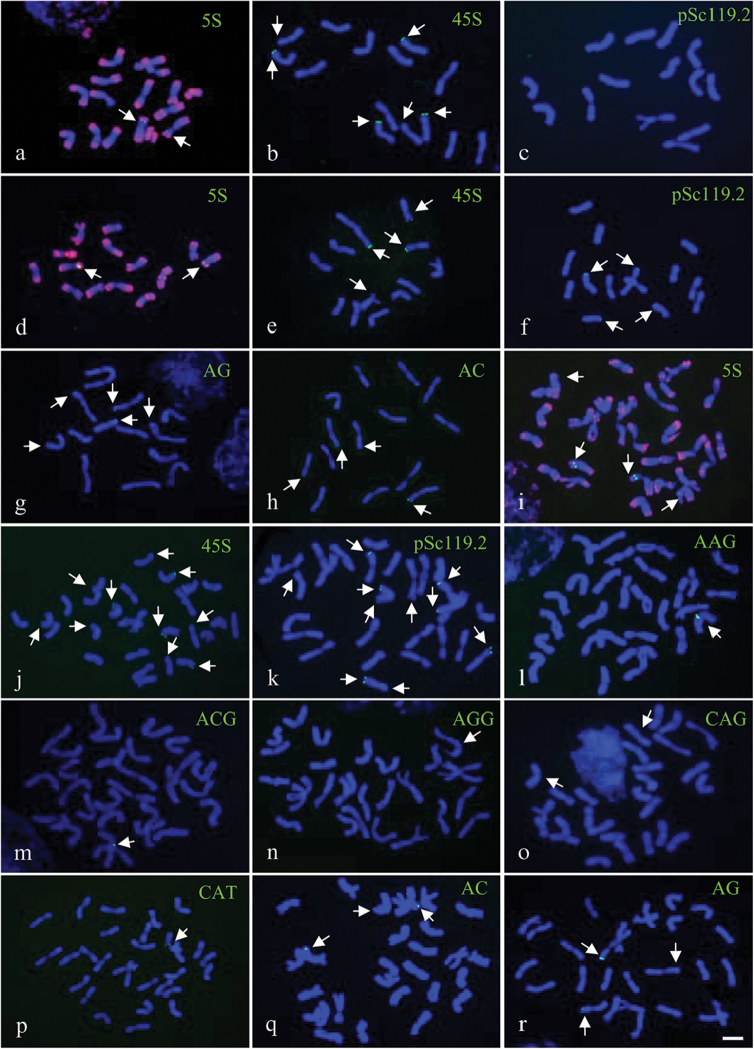
FISH patterns of mitotic metaphase chromosomes detected using pAs1 probes (red) combined with 5S rDNA, 45S rDNA, and pSc119.2 probes in *A. mongolicum* (**a–c**); 5S rDNA, 45S rDNA, pSc119.2, AG, and AC in *A. cristatum* (2x) (**d–h**); 5S rDNA, 45S rDNA, pSc119.2, AAG, ACG, AGG, CAG, CAT, AG, and AC in *A. cristatum* (4x) (**i–r**). The pAs1 signals (red) were removed artificially in all images except in (**a, d, i**). Arrows indicate the target signals (green). Bar = 10μm.

**Figure 2.**
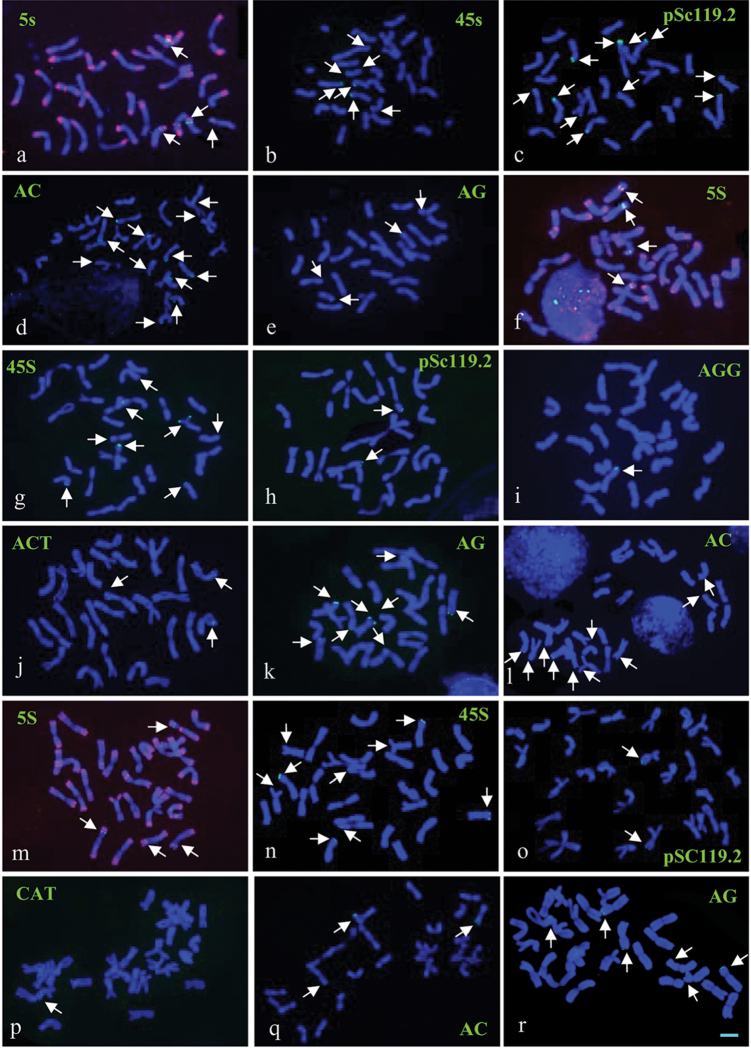
FISH patterns of mitotic metaphase chromosomes detected using pAs1 probes (red) combined with 5S rDNA, 45S rDNA, pSc119.2, AG, and AC probes in *A. desertorum* (**a–e**); 5S rDNA, 45S rDNA, pSc119.2, AAG, ACT, AG, and AC in *A. fragile* (**f–l**); 5S rDNA, 45S rDNA, pSc119.2, CAT, AC, and AG in *A. michnoi* (**m–r**). The pAs1 signals (red) were removed artificially in all pictures except in (**a, f, m**). Arrows indicate the target signals (green). Scale bar = 10 μm.

**Figure 3.**
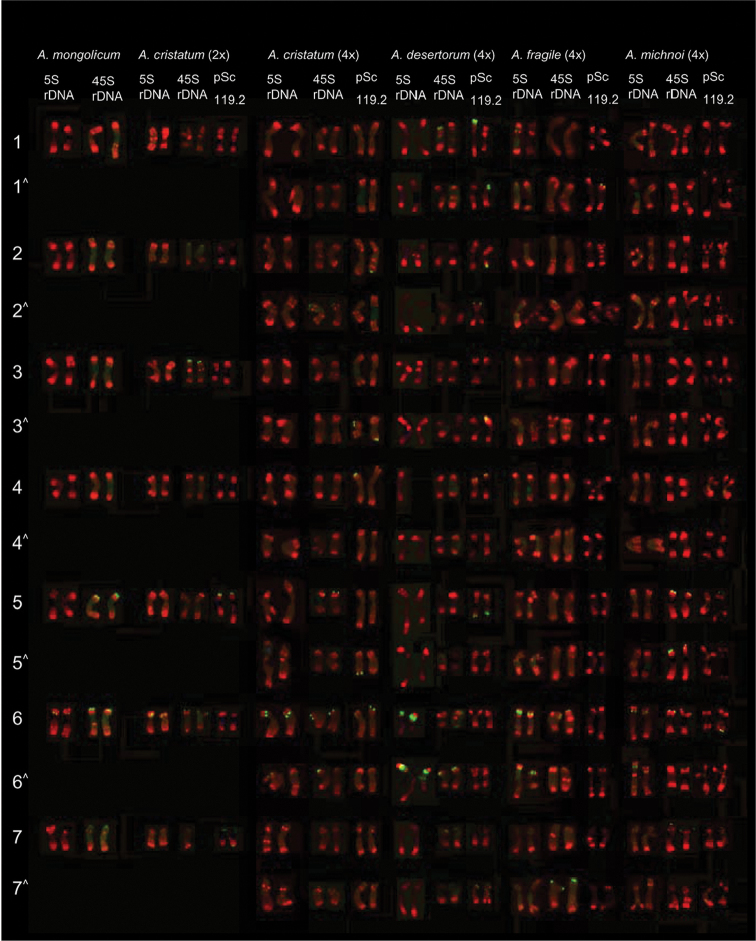
Molecular karyotypes of six *Agropyron* species probed using pAs1 (red) combined with 5S rDNA (green), 45S rDNA (green) or pSc119.2 (green) sequences. The numbers 1 to 7 designate seven different homologous chromosomes, whereas the same number followed by “^” indicates the respective homoeologous chromosomes in each genome of tetraploid species.

A chromosome number of 2n = 14 was detected in *A. mongolicum* (Fig. 1a–c) and *A. cristatum* (Fig. 1d–h), whereas a chromosome number of 2n = 28 was detected in most individuals of the other *Agropyron* species (Figs 1i–r, 2a–r). Both diploid and tetraploid species were detected. However, monosomic chromosomes were identified in many cases in tetraploid species (Figs 1o, r 2k, r). The repetitive sequence pAs1 showed multiple subtelomeric, intercalary, or pericentromeric hybridization sites on all chromosomes. The hybridization pattern of pAs1 was much more useful for distinguishing each chromosome of the P genome in all species compared with the other repetitive sequences. The tentative karyotypes of all individuals analyzed were determined using pAs1 as a land marker and the chromosome arm ratio and relative length as references (Fig. 3). Four pairs of metacentric chromosomes and three pairs of submetacentric chromosomes were identified in both *A. cristatum* and *A. mongolicum*. In addition, eight pairs of metacentric chromosomes and six pairs of submetacentric chromosomes were identified in the other tetraploid species. Although a high level of intra- and inter-species chromosomal polymorphisms were detected, homoeologous chromosomes among species were tentatively identified and designated 1-7 (1^^^-7^^^) (Fig. 3). In this designation, the numerals do not correspond with those of the homoeologous groups used in the nomenclature system of the chromosomes of wheat and barley. Seven homoeologous chromosomes were identified:

• Chromosome 1 is a metacentric chromosome with pAs1 hybridization signals in pericentromeric regions in most cases.

• Chromosome 2 is a metacentric chromosome lacking pAs1 hybridization signals in the subtelomeric region of the short arm in most cases.

• Chromosome 3 is a metacentric chromosome with pAs1 hybridization signals distributed from the intercalary to subtelomeric regions of both arms.

• Chromosome 4 is the smallest metacentric chromosome.

• Chromosome 5 is the largest submetacentric chromosome.

• Chromosome 6 is a submetacentric chromosome with the smallest arm ratio (short arm to long arm).

• Chromosome 7 is a submetacentric chromosome lacking pAs1 hybridization signals in the subtelomeric region of the short arm.

### Chromosomal distribution of 5S rDNA, 45S rDNA and pSc119.2 among species

The hybridization sites of 5S rDNA and 45S rDNA were stably detected in all analyzed species. The 5S rDNA hybridization signals were detected in one pair of chromosomes in the diploid species *A. mongolicum* and *A. cristatum* (Fig. 1a, d) and in two pairs of chromosomes in the other tetraploid species (Figs 1i, 2a, f, m). The physical position of the 5S rDNA sites were the most conserved, which were stably detected in the intercalary regions of the short arms of homoeologous chromosome 6 (6 and 6^^^ chromosomes). Notably, additional 5S rDNA sites in the pericentromeric regions of the long arms of chromosome 6^^^ and additional 5S rDNA sites in the intercalary regions of the short arms of chromosome 6^^^ were detected in *A. desertorum* and *A. fragile*, respectively (Fig. 3).

Four highly conserved 45S rDNA sites were stably revealed in subtelomeric regions in both diploid species (Fig. 1b and e). However, variable numbers of 45S rDNA sites (from four to six) were identified in tetraploid species: six for *A. cristatum* (Fig. 1j), five for *A. desertorum* (Fig. 2b), four for *A. fragile* (Fig. 2j) and five for *A. michnoi* (Fig. 2n). Most of the 45S rDNA sites were physically mapped to the subtelomeric regions of homoeologous chromosomes 2, 5, 6, and 7 among the tetraploid species. Occasionally, 45S rDNA sites were detected on chromosome 1 and in the subtelomeric regions of the long arms of chromosomes 4 and 6 in *A. desertorum* (Fig. 3). The 45S rDNA sites on homoeologous chromosome 6 were the most conserved, which were detected on both chromosomes 6 and 6^^^ in all tetraploid species. In addition, the 45 S rDNA sites on homoeologous chromosome 7 were only detected on one set of the homoeologous chromosomes (7 or 7^^^) (Fig. 3, Table 2).

A highly variable number of pSc119.2 hybridization sites was identified at either or both ends of the chromosomes in all diploid and tetraploid species, except *A. mongolicum*. The tetraploid species *A. cristatum* (Fig. 1k) and *A. desertorum* (Fig. 2c) harbored a high number of pSc119.2 hybridization sites (5–8; Table 2), whereas the other species carried as few as two sites. No conserved pSc119.2 sites were identified among the species.

### Chromosomal distribution of dinucleotide and trinucleotide SSRs among species

Among the four potential dinucleotide SSR probes, (AC)_15_, (AG)_15_, (AT)_15_, and (GC)_15,_ only (AC)_15_ and (AG)_15_ produced detectable hybridization signals, which were observed in subtelomeric regions, in all species analyzed, except *A. mongolicum* (Figs 1 and 2, Table 2). The number of hybridization signals for both (AC)_15_ and (AG)_15_ was highly among the deferent species. *Agropyron desertorum* (Fig. 2d) and *A. fragile* (Fig. 2l) carried a high number of (AC)_15_ hybridization signals (8–10), whereas the other species carried a low number of (AC)_15_ hybridization signals (1–3) (Table 2). Although the (AC)_15_ hybridization signals were frequently detected in homoeologous 1 chromosomes in most species, the conserved distribution of the (AC)_15_ signal among all investigated species was not observed. *Agropyron fragile* (Fig. 2k) and *A. michnoi* (Fig. 2r) carried a high number of (AG)_15_ hybridization signals (6–8), whereas the others carried low number of (AG)_15_ hybridization signals (3–4; Table 2).

Numbers followed by ” or ’ are the number of hybridization sites of the respective sequence. ” Indicates a pair of homologous chromosomes; ’ indicates one of the homologous chromosomes. Numbers in parentheses indicate the chromosome designation shown in Fig. 3

All ten potential trinucleotide SSRs probes, (AAG)_10_, (AAC)_10_, (AAT)_10_, (ACG)_10_, (ACT)_10_, (AGG)_10_, (CAC)_10_, (CAG)_10_, (CAT)_10_, and (GGC)_10_, were used to characterize the chromosomes of all species. No hybridization signals for any trinucleotide SSR probes were detected in the diploid species *A. mongolicum* and *A. cristatum* ‘Fairway’ or in the tetraploid species *A. desertorum*. A small number of SSR hybridization signals were identified in *A. cristatum* (4x), *A. fragile*, and *A. michnoi*, although these signals were not commonly shared among the species. *Agropyron cristatum* (4x) harbored the greatest number of trinucleotide SSR hybridization sites. One hybridization site of (AAG)_10_, (ACG)_10_, (AGG)_10_, (CAG)_10_ and (CAT)_10_ was well identified in the individuals of *A. cristatum* (4x) (Fig. 1l–p). All (AAG)_10_, (ACG)_10_, (CAG)_10_ and (CAT)_10_ sites were physically mapped to the pericentromeric regions of the long arms of chromosome 2. This finding implies the co-localized distribution of (AAG)_10_, (ACG)_10_, (CAG)_10_, and (CAT)_10_ in *A. cristatum* (4x). However, (AGG)_10_ was physically mapped to the pericentromeric region of the short arm of chromosome 5 (Table 2). Two trinucleotide SSRs (ACT)_10_ and (AGG)_10_, gave rise to hybridization signals in *A. fragile* (Fig. 2i and j). The SSR (ACT)_10_, which was not detected in the other species analyzed, was localized in the intercalary regions of the short arms of chromosome 4 in *A. fragile* (Fig. 2j).The (AGG)_10_ was hybridization site was physically mapped to subtelomeric regions in *A. fragile* (Fig. 2i) rather than to pericentromeric regions as in *A. cristatum* (4x) (Fig. 1n). Only one trinucleotide SSR, (CAT)_10_, was detected in *A. michnoi*. The (CAT)_10_ was hybridizatin site was localized to intercalary regions in *A. michnoi* (Fig. 2p) rather than to pericentromeric regions as in *A. cristatum* (4x) (Fig. 1p).

### B chromosome in *A. mongolicum* and *A. desertorum*

**Figure 4.**
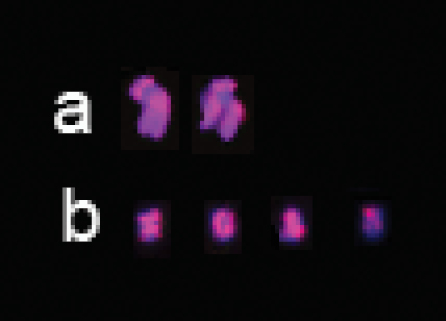
FISH patterns of B chromosomes detected by pAs1 (red). **a** submetacentric B chromosomes in *A. mongolicum* **b** metacentric B chromosomes in *A. desertorum*.

B chromosomes were identified in a few individuals of *A. mongolicum* (Fig. 1a) and *A. desertorum* (Fig. 2b) but not in the other *Agropyron* species analyzed. The B chromosomes in *A. mongolicum* and *A. desertorum* were intensely stained by the pAs1 probe. A distinct primary constriction was identified in the B chromosomes of both species. On the basis of the position of the primary constriction, the B chromosome in *A. mongolicum* was submetacentric, whereas that of *A. desertorum* was of metacentric type (Fig. 4).

**Table 2.** Number of hybridization sites and chromosomal distribution of the repetitive sequences for each *Agropyron* species.

Repeats	Species					
	*A. mongolicum*	*A. cristatum* (2x)	*A. cristatum* (4x)	*A. desertorum*	*A. fragile*	*A. michnoi*
**5S rDNA**	1” (6)	1” (6)	2” (6, 6^^^)	2” (6, 6^^^)	2” (6, 6^^^)	2” (6, 6^^^)
**45S rDNA**	4” (2, 5, 6, 7)	4” (2, 3, 6, 7)	6” (2^^^, 5, 5^^^, 6, 6^^^, 7^^^)	2”+3’ (6^^^, 7^^^+1, 4^^^, 6)	4” (2^^^, 6, 6^^^, 7^^^)	4”+1’ (5, 5^^^, 6, 7+6^^^)
**pSc119.2**	–	2” (5, 7)	4”+1” (3^^^, 4, 5^^^, 6^^^, +2)	3”+5’ (4, 5, 7^^^+1, 1^^^, 2, 2^^^, 3^^^)	2’ (1^^^, 6^^^)	2’ (5, 6)
**AC**	–	1’ (1)	1”+1’ (1+1^^^)	2”+4’ (1, 1^^^+ 2, 2^^^, 3, 4)	4”+2’ (1, 1^^^, 2, 2^^^+6, 7)	1”+1’ (4+ 6^^^)
**AG**	–	1”+1’ (5+2)	1”+1’ (1+3’)	4’ (2, 2^^^, 5, 7)	2”+4’ (3, 4^^^+ 2^^^, 3^^^, 5, 6)	2”+2’ (6, 2+2^^^, 3)
**AAG**	–	–	1’ (2)	–	–	–
**ACG**	–	–	1’ (2)	–	–	–
**ACT**	–	–	–	–	1”+1’ (4+4^^^)	–
**AGG**	–	–	1’ (5)	–	1’ (2)	–
**CAG**	–	–	1” (2)	–	–	–
**CAT**	–	–	1’ (2)	–	–	1’ (5)

## Discussion

### Genomic characterization of the P genome in Agropyron using repetitive sequences

In this study, all potential dinucleotide and trinucleotide SSRs and four satellite DNA repeats were used to characterize the P genome of five *Agropyron* species. Unlike other genomes such as the H, I, A, B, and D genomes in Triticeae (Cuadrado et al. 2007; 2008; Dou et al. 2016), which include a number of SSR hybridization signals based on FISH, the P genome in *Agropyron* harbored only the dinucleotide SSRs AG and AC in all species, except *A. mongolicum*, and fewer trinucleotide SSRs in the different species. The distribution of AAG corresponded to the distribution of N bands, which are composed of heterochromatin in Triticeae (Pedersen et al. 1996). In the P genome, only one AAG site was detected in the intercalary regions in one accession, *A. cristatum* (4x). The pAs1 in the P genome was detected more frequently in subtelomeric or pericentromeric regions than in intercalary regions, and the repeatedly detected 45S rDNA, pSc119.2, AC, and AG repeats were localized to subtelomeric regions. Forty-eight P-genome-specific sequences have been identified from the DOP-PCR product of the 6P chromosome of *A. cristatum*, and 14 of 48 sequences have been physically mapped to chromosomes, centromeres, pericentromeric regions, or subtelomeric regions throughout the P genome (Han et al. 2017). Taken together, the results of the present study suggest that the tandem-repetitive sequences in the P genome are much more intensively localized to the pericentromeric and subtelomeric regions rather than the intercalary regions.

The lack of tandem-repetitive sequences in intercalary regions makes the accurate discrimination of each chromosome of the P genome more difficult. In this study, 5S rDNA, 45S rDNA, and pAs1 repeats were more stable and informative. Four to five pairs or homoeologous chromosomes could be accurately identified in each diploid and tetraploid species based on the above-mentioned chromosomal markers, the chromosome length and the arm ratio.

### P genome differentiation and genetic relationships of the *Agropyron* species

The P genome of *A. mongolicum*, which includes no AG, AC or pSc119.2 repeats in the subtelomeric regions of any chromosome, is distinct from the P genome of the other *Agropyron* species analyzed. Structural rearrangements of some chromosomes were revealed between the two diploid species *A. cristatum* and *A. mongolicum* (Hsiao et al. 1986, 1989). In the present study, genome rearrangements were difficult to identify accurately, reflecting the low resolution of the chromosomal markers. However, discrepancies in the localization of the 45S rDNA sites were revealed on chromosomes 5 and 3 in *A. mongolicum* and *A. cristatum*, respectively. This observation strongly suggested that the chromosome structure arrangements involved these two chromosomes. The differentiation of both diploid species revealed in the present study suggests that the P genome of *A. cristatum* should be designated P^c^, and the P genome of *A. mongolicum* should be designated P^m^, consistent with the suggestions of Mellish et al. (2002). *Agropyron mongolicum* differs from other *Agropyron* species in producing a narrow, linear, glabrous spike (Dewey 1981). Data from AFLP markers discriminated *A. mongolicum* from the other *Agropyron* species (Mellish et al. 2002). Taken together, these results are strongly suggestive of a distant relationship between *A. mongolicum* and the other *Agropyron* species.

The tetraploid *A. cristatum* is considered to be an autopolyploid originating from the diploid species *A. cristatum*. However, the molecular karyotype of this tetraploid was not consistent with a doubled diploid karyotype. High variation in the number of hybridization sites and localization of 45S rDNA, AC, AG, and pSc119.2 repeats was revealed in the present study. Notably, a few trinucleotide SSRs were detected in tetraploid species rather than in diploid species. These observations indicate that high genomic variation, including DNA sequence deletion, amplification and genomic rearrangement, might have occurred in the transition from diploid to tetraploid species.

*Agropyron desertorum* is considered to be an allotetraploid between diploid *A. mongolicum* and *A. cristatum* (Asay et al. 1992, Mellish et al. 2002). The localization of a numer of 45S rDNA sites in *A. desertorum* was highly variable compared with the other species analyzed. Notably, one homoeologous set of chromosome 6 (6^^^) contained additional 5S rDNA sites in the pericentromeric regions of the long arm. The homoeologous chromosome 6 stably carried 5S rDNA sites in the short arms of all *Agropyron* species studied. The distinct differences in the FISH patterns between chromosomes 6 and 6^^^ strongly support the allopolyploid origin of *A. desertorum*. However, the FISH pattern of chromosome 6^^^ in *A. desertorum* was not similar to either *A. mongolicum* or *A. cristatum*.

*Agropyron fragile* has been suggested to be an autotetraploid derivative of *A. mongolicum* (Asay et al. 1992). The chromosomes of *A. fragile* include a few AG, AC, and pSc119.2 hybridization sites. The genomic characters of *A. fragile* are different from those of *A. mongolicum*. The present results did not support the suggestion that *A. fragile* is an autotetraploid derivative of *A. mongolicum*. Moreover, additional 5S rDNA sites were revealed in the intercalary regions of the short arms of one homoeologous set of chromosome 6 (6^^^). Similar to *A. desertorum*, the unique FISH pattern revealed in chromosome 6^^^ is supportive of a potential allopolyploid origin for *A. fragile*.

*Agropyron michnoi* exhibited a FISH pattern more similar to that of *A. cristatum* (4x). Specifically, the chromosomal co-linearity of the detected 45S rDNA sites was well retained between the two species, suggesting that *A. michnoi* might be more closely related to *A. cristatum* (4x) than to other *Agropyron* species studied.

### Variability of the repetitive sequences in the P genome

The repeats, such as AC, AG, and pSc119.2, that are localized in subtelomeric regions, showed a highly varying number of hybridizations among species with the P genome and within populations, particularly in tetraploid species. The presence or absence of hybridization signals might reflect the deletion or duplication of the repetitive sequences. Unequal crossing over is a type of gene duplication or deletion event (Graur et al. 2000). Highly variable repeats of the above-mentioned sequences in tetraploid species, which include two sets of the basic P genome, suggests that variability of repeats may be strongly driven by unequal crossing over during the interfered meiosis process in tetraploid species with two similar genomes. The small number of trinucleotide SSRs hybridization sites and aneuploids frequently detected in tetraploid species might further confirm interference of meiosis in tetraploid species.

Specific FISH patterns of trinucleotide SSRs were detected in three different *Agropyron* species. Extensive intra- or inter-population genetic variation has been detected in *Agropyron* species (Mellish et al. 2002, Che et al. 2011, Chen et al. 2013). However, limited materials of each species were analyzed in the present study. Whether the FISH patterns of trinucleotide SSRs also exhibit high intraspecific variation needs further investigation.
